# Obg-like ATPase 1 (OLA1) overexpression predicts poor prognosis and promotes tumor progression by regulating P21/CDK2 in hepatocellular carcinoma

**DOI:** 10.18632/aging.102797

**Published:** 2020-02-11

**Authors:** Shanzhou Huang, Chuanzhao Zhang, Chengjun Sun, Yuchen Hou, Yixi Zhang, Nga Lei Tam, Zekang Wang, Jia Yu, Bowen Huang, Hongkai Zhuang, Zixuan Zhou, Zuyi Ma, Zhonghai Sun, Xiaoshun He, Qi Zhou, Baohua Hou, Linwei Wu

**Affiliations:** 1 Organ Transplant Center, The First Affiliated Hospital, Sun Yat-Sen University, Guangzhou 510080, China; 2 Department of General Surgery, Guangdong Provincial People's Hospital, Guangdong Academy of Medical Sciences, School of Medicine, South China University of Technology, Guangzhou 510080, China; 3 The Fifth Affiliated Hospital of Sun Yat-Sen University, Division of Hepatobiliary Surgery, Zhuhai 519000, China; 4 Department of Liver Surgery, The First Affiliated Hospital of Sun Yat-Sen University, Guangzhou 510080, China; 5 China Department of General Surgery, Hui Ya Hospital of The First Affiliated Hospital, Sun Yat-Sen University, Huizhou, Guangdong 516081, China

**Keywords:** HCC, OAL1, CDK2, prognosis, tumor progression

## Abstract

Background: Obg-like ATPase 1 (OLA1) has been found to have a dual role in cancers. However, the relationship between OLA1 and hepatocellular carcinoma (HCC) remains unclear.

Results: High expression of OLA1 in HCC was detected in public datasets and clinical samples, and correlated with poor prognosis. Downregulation of OLA1 significantly inhibited the proliferation, migration, invasion and tumorigenicity of HCC cells. Mechanistically, GSEA showed that OLA1 might promote tumor progression by regulating the cell cycle and apoptosis. In addition, OLA1 knockdown resulted in G0/G1 phase arrest and high levels of apoptosis. OLA1 could bind with P21 and upregulate CDK2 expression to promote HCC progression.

Conclusions: Overall, these findings uncover a role for OLA1 in regulating the proliferation and apoptosis of HCC cells.

Materials and methods: The Cancer Genome Atlas and Gene Expression Omnibus datasets were analyzed to identify gene expression. Immunohistochemistry staining, western blot and real-time polymerase chain reaction were performed to evaluate OLA1 expression in samples. Cell count Kit-8, wound-healing, transwell and flow cytometry assays were used to analyze HCC cell progression. Subcutaneous xenotransplantation models were used to investigate the role of OLA1 in vivo. Coimmunoprecipitation was used to analyze protein interactions.

## INTRODUCTION

Hepatocellular carcinoma (HCC), a highly aggressive malignancy, is one of the most common cancers and fatal malignancies worldwide [1, 2]. Despite the great advances in treatment approaches that have been achieved, the long-term outcome for HCC patients remains unsatisfactory because of its high recurrence and metastasis rate [3, 4]. Over the past several decades, the incidence of HCC has decreased, while the mortality rate has increased in both the male and female populations [5]. Most HCC cases develop from viral hepatitis, alcoholism, or metabolic disorders [6]. HCC is most prevalent in Eastern Asia because of chronic infection with hepatitis B virus (HBV) [3, 7, 8]. Currently, important insights into the biology of HCC have been gained by omics studies [9, 10]. Studies have provided insights into the tumor biology of HCC and suggest opportunities for personalized therapies [11]. However, the detailed mechanism underlying HCC is lacking. Therefore, exploration of HCC progression to develop new therapeutic targets is especially urgent.

Obg-like ATPase 1 (OLA1) was found to be vital for the biosynthesis of cytoplasmic protein in yeast, Saccharomyces cerevisiae [12]. It plays a dual role in cell survival, growth and progression by binding to eukaryotic initiation factor 2 (E2F) [13]. Wenk et al. [14] also reported that OLA1 most likely executes similar functions in bacteria and humans, and its upregulation inhibits the ability of the cells to scavenge damaging reactive oxygen species. Moreover, OLA1 appears to influence cell proliferation and was found to be upregulated in many tumors. OLA1 overexpression has been detected in multiple types of cancer and may be related to poor survival. OLA1 mediates the phosphorylation of serine and threonine on proteins in cancer cells by restraining the GSK3β-inhibitor 2-PP1 positive feedback loop, leading to more aggressive tumor growth [15]. With knockdown of OLA1, cell migration and invasion in breast cancer cells were significantly inhibited via the modulation of intracellular oxidative stress levels [16]. OLA1 was also found to be a functional protein in regulating cell-matrix adhesion, and it could lead to significant changes in cell adhesion and the associated phenotypes [17]. The encoded protein interacts with breast cancer-associated gene 1 (BRCA1) and BRCA1-associated RING domain protein (BARD1), resulting in centrosome regulation [18, 19]. However, the role of OLA1 in HCC remains unknown. Herein, we found an elevated expression of OLA1 in HCC and further explored the underlying mechanism for the first time.

In mammalian cells, the G1-to-S phase transition requires the expression of G1 cyclins D and E and the formation and activation of the cyclin D-CDK4/6 and cyclin E-CDK2 complexes, which phosphorylate and inactivate Rb to release E2F, allowing E2F-mediated transcriptional activity. Then, the cell cycle will enter S-phase [20, 21]. The G2-to-M transition requires the activation of the cyclin B-CDK1 complex via the CDC25-mediated dephosphorylation of CDK1 [22]. The G1/S and G2/M transitions are also negatively regulated by cyclin-dependent kinase inhibitors (CKIs), such as P21 [23]. As has been widely acknowledged, P21 plays an important role in regulating the cell cycle, repairing damaged DNA, scavenging free radicals and regulating the immune response. CDK2 promotes S phase initiation of the cell cycle via the formation of functional complexes with cyclin A and cyclin E [24]. Upregulation of CDK2 expression can be found in various solid tumors and is closely related to the formation and development of tumors [25–27]. DNA damage upregulates the P21 protein, which can bind to and inhibit the CDK2 kinase [28].

In our study, we found that OLA1 was significantly upregulated in HCC patients and was closely related to poor survival and malignant clinical characteristics. Knockdown of OLA1 inhibited HCC progression and tumorigenicity, which at the molecular level was associated with reduced G1/S-specific cyclins and increased levels of the cyclin-dependent kinase inhibitor (CKI) P21. The results of our study provide evidence that OLA1 might be a vital biomarker for diagnosis and a potential target for treating HCC.

## RESULTS

### OLA1 is upregulated in HCC and correlates with unfavorable clinical characteristics

**Figure 1:**
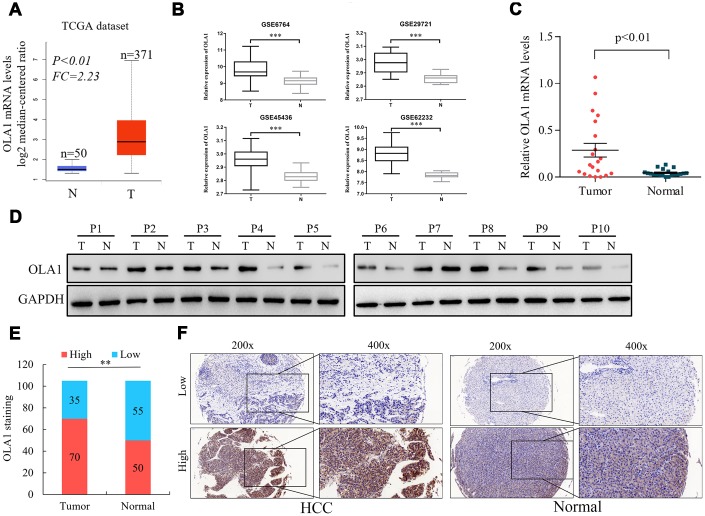
**Upregulation of OLA1 in hepatocellular carcinoma (HCC).** (**A**) The Cancer Genome Atlas (TCGA) liver cancer dataset (liver hepatocellular carcinoma) included the messenger RNA (mRNA) expression of OLA1 in HCC tissues (n = 371) and normal tissues (n = 50). (**B**) OLA1 was highly expressed in HCC tissues compared with normal liver tissues according to the analysis of GEO datasets. GSE6764, tumor, n=35; normal, n=23. GSE29721, tumor, n=10; normal, n=10. GSE45436, tumor, n=95; normal, n=39. GSE62232, tumor, n=81; normal, n=10. (**C**) Real-time PCR analysis of OLA1 expression in 20 pairs of HCC specimens and corresponding normal liver tissues (P<0.01). (**D**) OLA1 is upregulated in HCC tissues, as shown by western blot results. (**E**) OLA1 is upregulated in 70 out of 105 HCC tissues, as shown by IHC. (**F**) Representative images of OLA1 staining in HCC specimens and normal liver tissues. P, patient; T, tumor; N, normal.

To assess the role of OLA1 in the progression of HCC, we employed accessible human HCC datasets from the TCGA database and the GEO datable (GSE6764, GSE29721, GSE45436 and GSE62232). The results revealed that OLA1 was significantly elevated in HCC tissues among all data sets compared to normal liver tissues (Figure 1A and 1B). Real-time PCR analysis of 20 pairs of fresh tumor tissues and normal liver tissues demonstrated that OLA1 was upregulated in HCC tissues (Figure 1C, p<0.01). As expected, OLA1 was significantly increased in HCC tissues compared with in normal control tissues at the protein level (Figure 1D). Immunohistochemistry (IHC) results confirmed that 70 out of 105 HCC patients showed high levels of OLA1 expression, while 35 had low levels of OLA1 expression (Figure 1E and 1F). OLA1 was not upregulated in normal liver tissues as shown by IHC (Figure 1D and 1F).

### Upregulation of OLA1 correlates with unfavorable clinical characteristics and predicts poor survival in HCC patients

**Figure 2:**
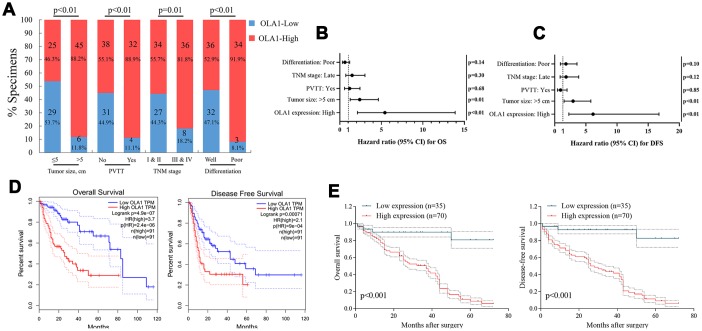
**OLA1 expression is correlated with clinicopathological features and poor prognosis.** (**A**) OLA1 expression levels were significantly correlated with tumor size, PVTT, TNM stage and tumor differentiation. (**B** and **C**) OLA1 expression and several other clinical characteristics were analyzed by multivariate Cox regression analysis to determine whether they were independent prognostic factors in predicting the overall survival (OS) and disease-free survival (DFS) of HCC patients. (**D**) Overall survival and disease-free survival analysis of TCGA data. (**E**) Overall survival and disease-free survival curves for HCC patient groups seen in our clinical data.

The clinical significance of high OLA1 expression was further assessed by IHC staining in 105 HCC tissue samples and adjacent normal liver tissues (Supplementary Table 1). The correlation between OLA1 expression and the clinical features of hepatocellular carcinoma is shown in Figure 2A and Supplementary Tables 2 and 3. OLA1 expression was positively correlated with tumor size (p<0.01), PVTT (p<0.01), TNM stage (p=0.01) and tumor differentiation degree (p<0.01). The expression of OLA1 was upregulated in HCC and positively correlated with the malignancy of HCC. In the multivariate Cox regression models, OLA1 was an independent prognostic factor for the overall survival (OS) and disease-free survival (DFS) of 105 HCC patients from our center (Figure 2B and 2C). To determine the prognostic value of OLA1, we performed Kaplan-Meier analysis. Overexpression of OLA1 was associated with inferior OS and DFS in both the TCGA cohort (Figure 2D) and HCC patients at our center (Figure 2E). As indicated by the clinical outcomes, OLA1 can be used as an independent biomarker for the prognosis of HCC patients.

### OLA1 promotes the progression and tumorigenicity of HCC cells

**Figure 3:**
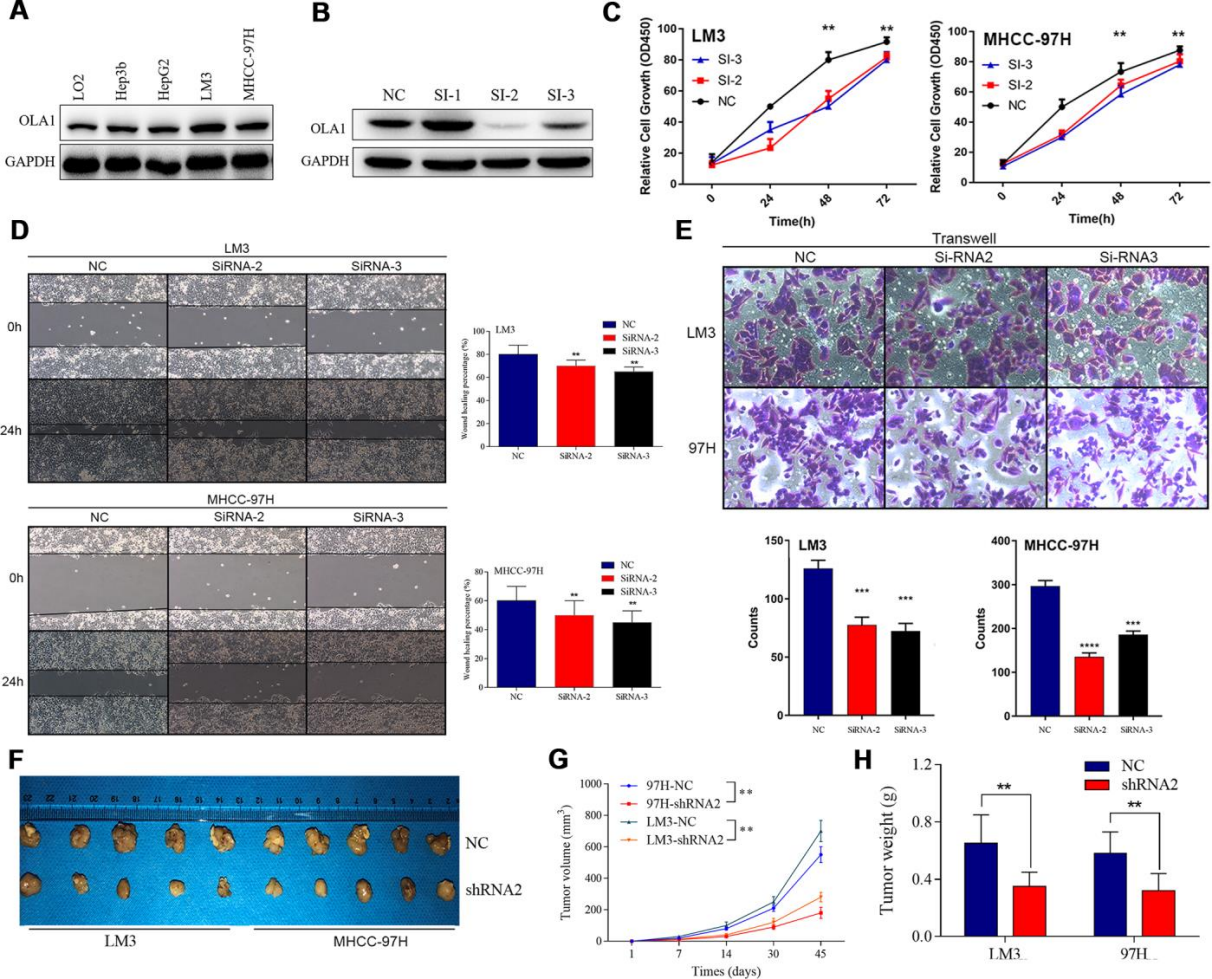
**Inhibition of OLA1 represses the proliferation, migration, invasion and tumorigenicity of HCC cells.** (**A** and **B**) OLA1 was highly expressed in LM3 and MHCC-97H cells and inhibited by transfection with OLA1-siRNA. (**C**) OLA1 silencing repressed the proliferation of HCC cells. (**D**) OLA1 downregulation inhibited cell migration after transfection. (**E**) The transwell Matrigel penetration assay showed that OLA1 promoted the invasion of HCC cells. (**F**–**H**) OLA1 silencing significantly reduced tumor growth and tumor weight in xenografts carrying HCC cells. (**I**) Ki-67 was significantly downregulated in OLA1-knockout tumors, as shown by IHC. All *p<0.05, **p<0.01, ***p<0.001.

We detected the expression of OLA1 in HCC cell lines and found that OLA1 was highly expressed in LM3 and MHCC-97H cells (Figure 3A). The biological role of OLA1 was further investigated in HCC. To study the role of OLA1 in HCC, three different siRNAs targeting OLA1 (siRNA-1, siRNA-2 and siRNA-3) were transfected into the LM3 and MHCC-97H cell lines. Western blot analysis showed that the expression of OLA1 in the cells transfected with OLA1-siRNA was significantly lower than that in the control group (Figure 3B). With the silencing of OLA1, proliferation was repressed, as indicated by the MTT assay results (Figure 3C). Moreover, downregulation of OLA1 in LM3 and MHCC-97H cells inhibited cell migration, as shown by the wound-healing assay results (Figure 3D). The transwell Matrigel penetration assay showed that OLA1 promoted the invasion of HCC cells (Figure 3E). To detect whether OLA1 expression levels affected tumor progression in vivo, we constructed stable OLA1-knockout LM3 and MHCC-97H cells using lentiviruses carrying shRNA. Silencing of OLA1 significantly inhibited the growth and weight of both LM3 and MHCC-97H tumors (Figure 3F–3H).

### OLA1 participates in cell cycle regulation in HCC cells

**Figure 4:**
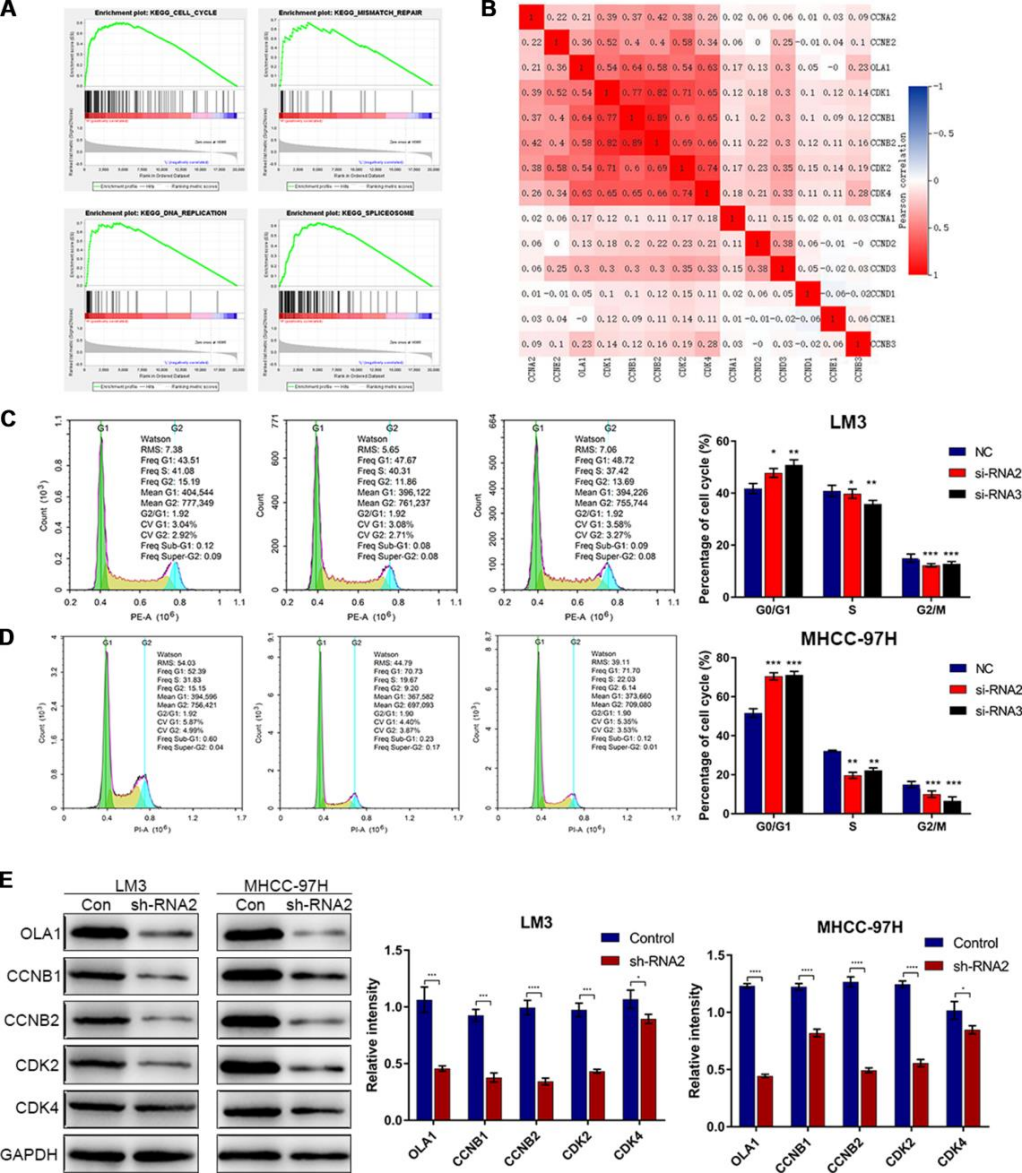
**Downregulation of OLA1 arrests the cell cycle in HCC cells.** (**A**) GSEA using TCGA datasets suggested that OLA1 is related to the cell cycle in HCC. (**B**) Correlation analysis showed that OLA1 is strongly related to CDK1, CDK2, CDK4, CCNB1 and CCNB2. (**C** and **D**) Depletion of OLA1 triggered G0/G1 blockade in LM3 and MHCC-97H cells. (**E**) Western blot analysis suggested that knockdown of OLA1 downregulated CDK1, CDK2, CDK4, CCNB1 and CCNB2. All *P<0.05, **P<0.01, ***P<0.001, ****P<0.0001.

To identify the specific function of OLA1 in HCC, we performed GSEA between low and high OLA1 expression datasets. We selected the most significantly enriched signaling pathways based on their normalized enrichment scores. GSEA showed that the terms cell cycle pathway, mismatch repair, DNA replication and spliceosome were the most differentially enriched in the OLA1-high expression group (Figure 4A). Then, we used TCGA data to analyze the relationships among OLA1, cyclins and CDKs. The expression levels of CDK1, CDK2, CDK4, CCNB1 and CCNB2 were positively correlated with OLA1 (Pearson correlation coefficient r^2^≥0.54) (Figure 4B). We further studied the effect of OLA1 on the cell cycle by flow cytometry. As shown in Figure 4C and 4D, the cell cycle of the OLA1 siRNA groups was arrested at the G0 phase (p< 0.05). Moreover, western blot results showed that the protein levels of CDK2, CDK4, CCNB1 and CCNB2 were lower in sh-RNA2-treated cells than in cells of the control group (Figure 4E). OLA1 is strongly associated with cell cycle regulation in HCC.

### Knockdown of OLA1 promotes apoptosis

**Figure 5:**
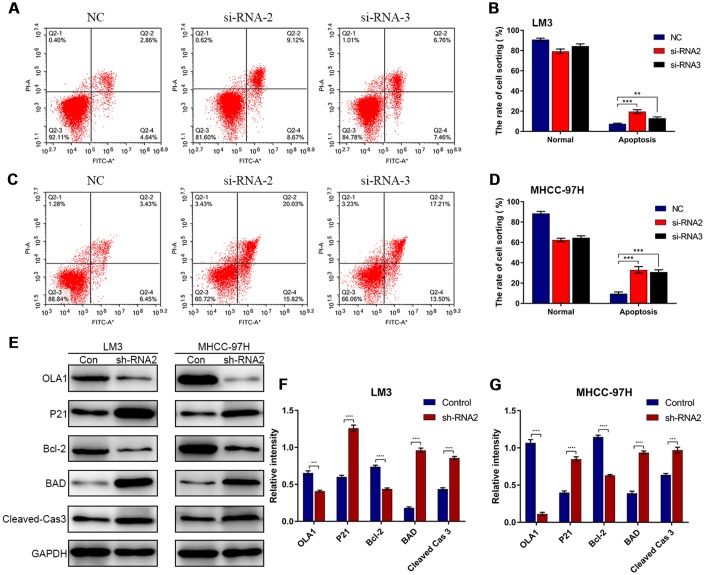
**Depletion of OLA1 promotes apoptosis in HCC cells.** (**A**–**D**) Flow cytometry was performed to determine the level of cell apoptosis in si-OLA1 groups. (**E**–**G**) Western blot analysis showed that knockdown of OLA1 upregulated P21, BAD and cleaved caspase 3 levels and downregulated Bcl-2 levels in HCC cells. All *P<0.05, **P<0.01, ***P<0.001, ****P<0.0001.

Flow cytometry results showed that depletion of OLA1 increased the percentage of apoptotic LM3 and MHCC-97H cells (Figure 5A–5D). Western blot results showed that the expression of P21, BAD and cleaved caspase 3 was elevated in sh-OLA1 HCC cells (Figure 5E–5G). Moreover, depletion of OLA1 significantly inhibited Bcl-2 in LM3 and MHCC-97H cells (Figure 5E–5G). OLA1 could promote apoptosis resistance in HCC by decreasing BAD/P21/cleaved caspase 3 levels.

### OLA1 activates the HCC cell cycle by inhibiting the interaction between P21 and CDK2

**Figure 6:**
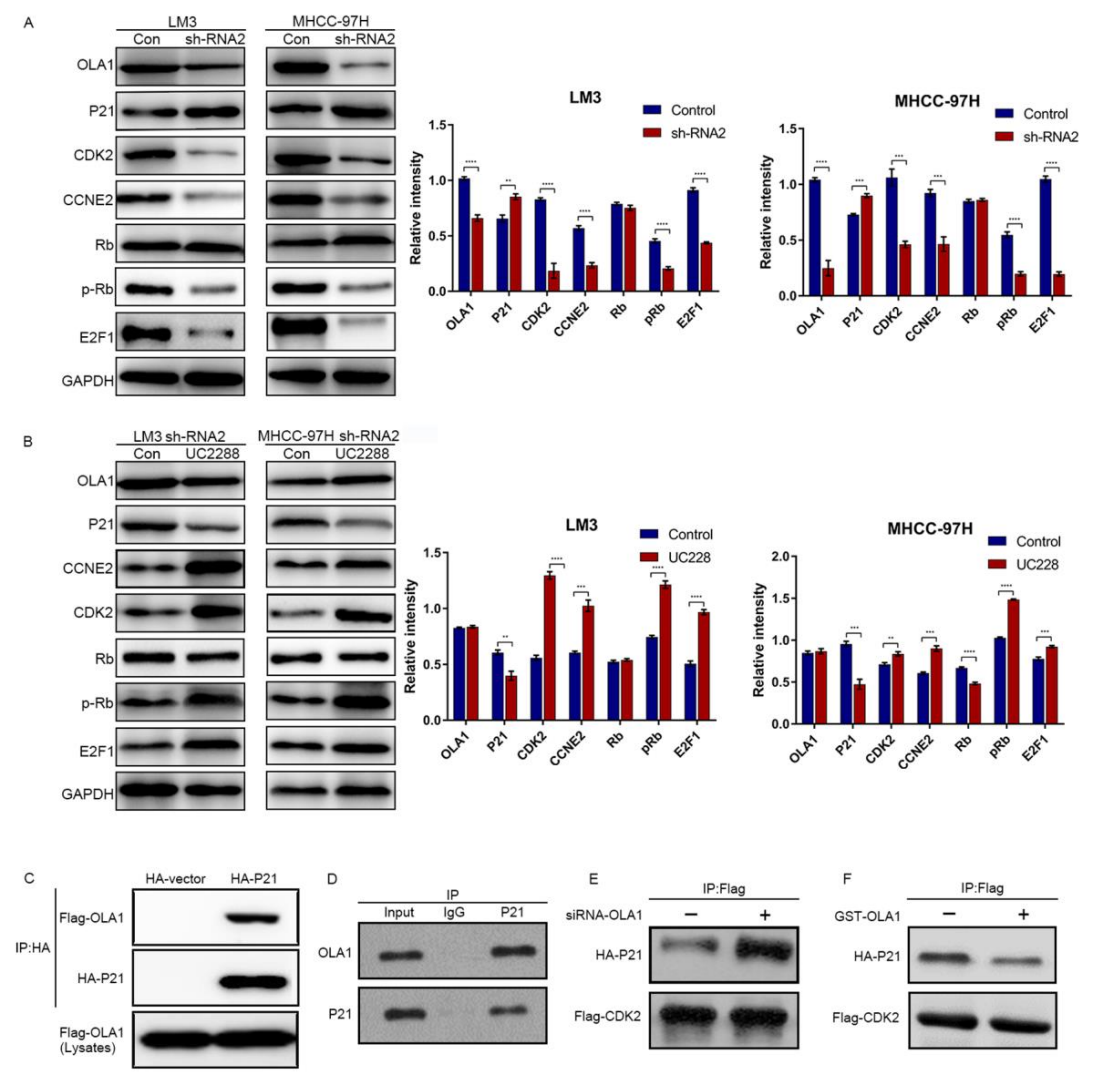
**OLA1 inhibits the interaction between CDK2 and P21.** (**A**) LM3 and MHCC-97H cells were transfected with OLA1 sh-RNA2 or treated with the P21 inhibitor UC2288 for 48 h, and cell lysates were analyzed by western blot using the indicated antibodies. (**B**) HCC cells were transfected with Flag-CDK2 and HA-P21 in the presence or absence of siRNA-OLA1 or GST-OLA1 for 48 h. Flag-CDK2 and HA-P21 were examined by co-IP assays, followed by western blot. (**C**) HEK293T cells were transfected with Flag-OLA1 or HA-P21 (or both) for 48 h. The interaction between exogenous OLA1 and P21 was examined by co-IP assays, followed by western blot. (**D**) The interaction between endogenous OLA1 and P21 was examined by co-IP assays, followed by western blot. h, hour.

The above results demonstrated that OLA1 regulates the cell cycle and inhibits cell apoptosis. Next, we aimed to study the specific underlying mechanism. Western blot analysis showed that depletion of OLA1 upregulated P21 levels and reduced the phosphorylation of Rb (Figure 6A). With OLA1 depletion, CDK2, CCNE2, and E2F1 levels were significantly repressed in HCC cells. After using an inhibitor of P21 UC2288 in HCC OLA1-knockdown cells, western blot results showed that CCNE2, CDK2, E2F1 and phosphorylated Rb levels were upregulated (Figure 6A). To further investigate the correlations between OLA1, CDK2 and P21, we performed coimmunoprecipitation (co-IP) experiments. In endogenous level of HCC cells, with OLA1 downregulation, less OLA1 interacts with P21, resulting in more P21 being pulled down by Flag-CDK2 (Figure 6B). With OLA1 upregulation, more OLA1 interacts with P21 resulting less P21 binding to CDK2. CDK2 level was not regulated due to OLA1 level (Figure 6B). OLA1 protein can interact with P21 protein directly with overexpression system in 293T cells (Figure 6C and 6D). These results suggested that OLA1 inhibited the phosphorylation of Rb by interfering with the interaction between CDK2 and P21.

### OLA1 correlates with CDK2 in HCC

**Figure 7:**
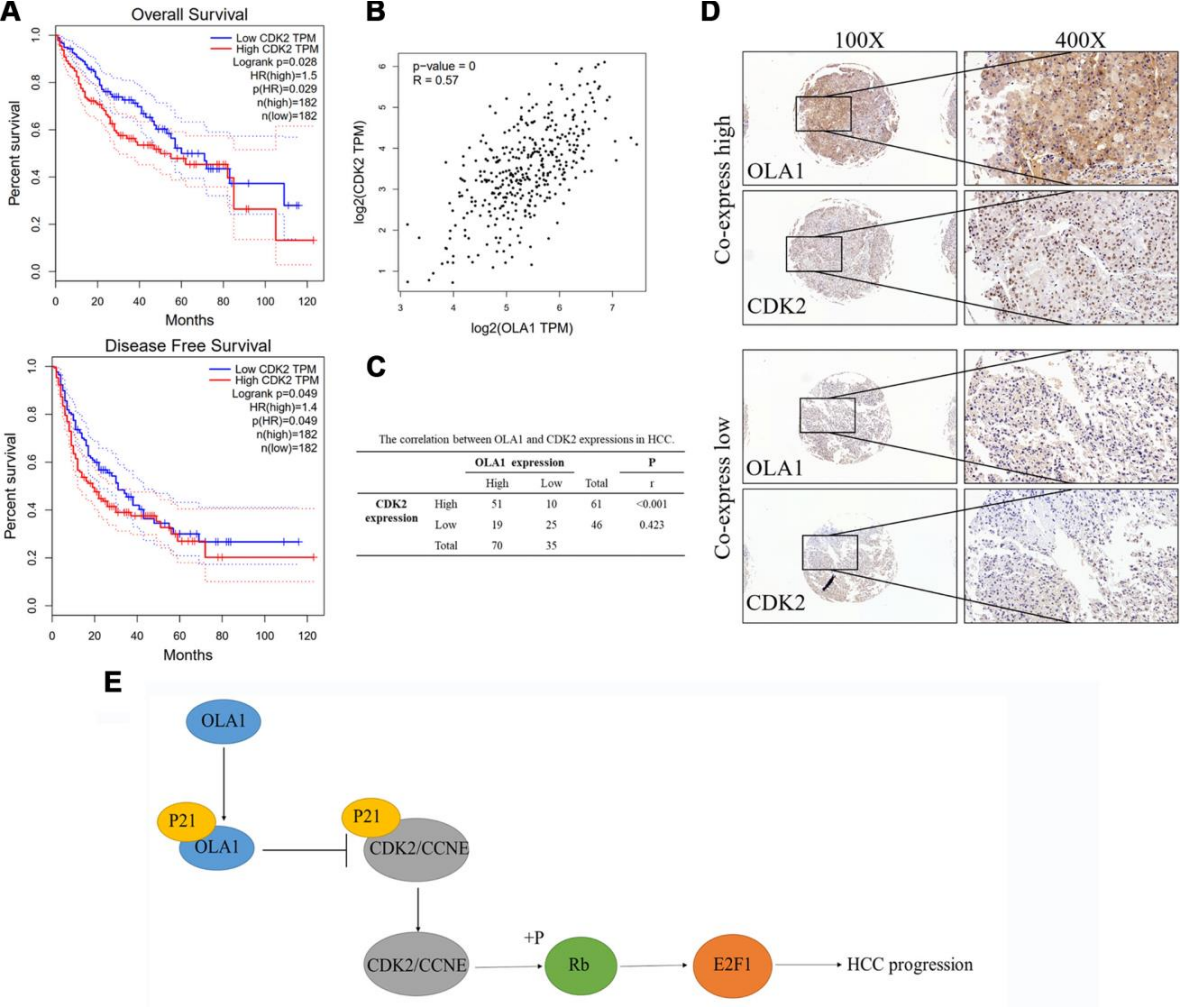
**OLA1 correlates with CDK2 in HCC.** (**A**) High expression of CDK2 was associated with poor survival outcomes of HCC. (**B**) CDK2 expression level is highly relevant to OLA1 expression. (**C**) Our HCC clinical samples also demonstrated a high correlation of OLA1 and CDK2. (**D**) Representative images of OLA1 and CDK2 co-expression staining in HCC specimens. (**E**) Schematic of the above-mentioned findings. OLA1 influences cell cycle, apoptosis and progression in LM3 and MHCC-97H cells by the interaction with P21 and upregulation of CDK2.

High expression of CDK2 was associated with poor survival outcomes of HCC as indicated by TCGA data (Figure 7A). Further, the expression level of CDK2 is highly relevant to the level of OLA1 (R = 0.57, p<0.001, Figure 7B). Our HCC clinical samples also demonstrated a high correlation of these biomarkers (Figure 7C and 7D).

## DISCUSSION

There has not been satisfactory improvement in the long-term survival of HCC patients [29]. Therefore, developing novel treatment targets and uncovering the underlying molecular mechanism have become increasingly urgent. We performed the first study of OLA1 in HCC. In the present study, OLA1 serves as a marker for predicting inferior prognosis in HCC patients. High OLA1 expression was therefore identified as an independent prognostic factor for the prediction of OS and DFS in HCC. Our data showed that OLA1 facilitated the growth, migration, invasion, apoptosis resistance and tumorigenesis of HCC cells (Figures 3–6 and Supplementary Figure 1). Mechanistic studies have suggested that OLA1 activates tumorigenicity and progression in HCC by regulating CDK2 and P21 to drive the activation of the Rb/E2F1 pathway.

OLA1 is a member of a subfamily of P-loop GTPases [30]. P-loop GTPases are the most abundant nucleotide-binding proteins and are involved in the regulation of diverse cellular processes, including protein translation, intracellular transport, signal transduction, cell proliferation, and stress response [28]. A novel molecular mechanism by which OLA1 stabilizes HSP70, leading to the upregulation of HSP70 and increased survival during heat shock, has been reported [31]. Zhang et al. [32] found that knockdown of OLA1 in human cells elicited an increased resistance to oxidizing agents, including tert-butyl hydroperoxide and diamide, without affecting cell proliferation, baseline apoptosis, or sensitivity to other cytotoxic agents that target the mitochondria, cytoskeleton, or DNA. Studies have revealed that the modulation of OLA1 in tumorigenesis may be two-sided, either inhibitory or promoting the cancer. One previous report indicated that OLA1 is overexpressed in colon, stomach, ovarian, uterine, and lung cancers at the mRNA level and in colon cancer at the protein level compared to its expression in normal tissue counterparts [33]. Bai et al. [34] found that decreased OLA1 protein expression was associated with an increased risk of relapse and decreased disease-specific survival, indicating that OLA1 may play a cancer type-specific role in cancer progression. In the experimental EMT system, Bai et al. [34] found that OLA1-knockdown lung cancer cells were more resistant to TGF-β1-induced EMT than the control-siRNA transfected cells. In breast and ovarian cancer cells, OLA1 directly bound to the amino-terminal region of BRCA1 and γ-tubulin, resulting in centrosome regulation [18]. A further study showed that receptor for activated C kinase was a protein that interacted with OLA1 and controlled the localization of BRCA1 in the centrosome, resulting in regulation of centriole duplication in mammary tissue-derived cells [35]. As shown by previous studies, OLA1 functioned differently in various conditions, especially in different phases of the cell cycle [36]. The results demonstrate that OLA1 has a vital role in promoting the progression of HCC and could serve as a therapeutic target.

To investigate how OLA1 functioned in regulating cell proliferation and progression, we explored the possibility that OLA1 might interact with cell cycle-associated proteins (Figures 4–6). In the present study, bioinformatics analysis found that OLA1 had a close relationship with CDK2. CDK2 binding to cyclin E initiates transition from the G1 to the S phase. This interaction can be inhibited by P21 binding to CDK2 [26]. Our results showed that OLA1 interacted with both P21 and free CDK2. Formation of the OLA1–P21 complex upregulated CDK2 expression. Next, CDK2 binds to cyclin E or cyclin A, and CDK2 has been shown to be active in the G1/S and M phases [37, 38]. Figure 6 shows that the presence of free CDK2 will result in Rb phosphorylation and CCNE2 and E2F1 upregulation, thus processing the transition from G1 to S phase. After knockdown of OLA1, CDK2 was significantly downregulated (Figure 4). As a result, the initiation of the transition from G1 to S phase was inhibited, which explained the G1 arrest of HCC cells following OLA1 knockdown.

Cancer represents a pathological manifestation of uncontrolled cell division and cell cycle mis-regulation. Therefore, it has long been anticipated that our understanding of the basic principles of cell cycle control would result in effective cancer therapies [25]. In particular, CDKs that promote transitions throughout the cell cycle were expected to be key therapeutic targets [39]. Upon DNA damage or other stressors, the tumor suppressor p53 is activated, leading to transient expression of the CDK inhibitor P21. This either triggers momentary G1 cell cycle arrest or leads to a chronic state of senescence or apoptosis, a form of genome guardianship [40]. P21 is involved in differentiation, cell migration, cytoskeletal dynamics, apoptosis, transcription, DNA repair, reprogramming of induced pluripotent stem cells, autophagy and the onset of senescence [41]. Therefore, P21 is considered to be one of the key factors determining cell survival and the focus of novel treatment strategies [42]. Here, we first found that OLA1 could bind to P21 in HCC, thereby inhibiting P21 transcriptional activity and the P21-mediated inhibition of HCC cell progression. After knocking down OLA1, the expression of CDK2 was greatly reduced. Therefore, we believe that OLA1 binds to and inhibits P21 to promote CDK2. In addition, we found that OLA1 interacted with P21 and CDK2 by immunoprecipitation experiments. The interactions of OLA1, CDK2 and P21 greatly affect the cell cycle and promote HCC progression.

In conclusion, we demonstrated that increased OLA1 expression is an unfavorable marker in HCC survival. OLA1 regulated proliferation, migration, invasion, apoptosis, the cell cycle and tumorigenicity in HCC cells. In-depth mechanistic studies suggested that OLA1 binds to P21, resulting in increased expression of CDK2, followed by phosphorylation of Rb and an increase in E2F1 (Figure 7E). Collectively, our present study provides novel insights into the mechanism of tumorigenesis in HCC, as well as a vital biomarker for diagnosis and a potential target for the treatment of HCC.

## MATERIALS AND METHODS

### Patients, tissue samples and follow-up

All tissue samples were obtained from HCC patients who underwent hepatectomy at the First Affiliated Hospital of Sun Yat-sen University from July 2013 to December 2015 [43]. Preoperative radiotherapy or preoperative chemotherapy was not performed. Detailed clinical information of all patients is shown in the extended data. All fresh tumor tissue specimens for mRNA detection were cryopreserved rapidly in liquid nitrogen and stored immediately at -80°C after resection. All patients were followed up until July 2019. Overall survival (OS) was defined as the period between liver resection and death or the last contact. Disease-free survival (DFS) was defined as the period between liver resection and any form of tumor recurrence or metastasis [44].

### High-throughput data processing

To investigate the clinical significance of OLA1 in HCC, data from samples from the TCGA database (https://gdc.cancer.gov/) and the GEO database (GSE6764, GSE29721, GSE45436 and GSE62232) (http://www.ncbi.nlm.nih.gov/geo) were analyzed. All data were Log2 converted and analyzed by R and GraphPad Prism 8 software. The relative expression levels were determined. The edgeR package that we used was based on negative binomial distributions, an empirical Bayes estimation, exact tests, generalized linear models and quasi-likelihood tests. A logFC (fold change) ≥1.0 or a logFC ≤-1.0 associated with a P value <0.05 was selected as statistically significant for the genes.

### GSEA

GSEA was performed to identify gene sets, pathways, and OS and DFS outcomes associated with OLA1 using the data obtained from TCGA. In this study, GSEA first generated an ordered list of all genes according to their correlation with OLA1 expression. GSEA was carried out to elucidate the significant survival differences observed between the high- and low-OLA1 groups. Gene set permutations were performed 1,000 times for each analysis. The expression level of OLA1 was used as a phenotype label. GSEA was carried out via the Broad Institute Website (http://software.broadinstitute.org/gsea/index.jsp). Each gene in the list was weighted by its log fold change in expression.

### Cell culture and transfection

Human HCC cell lines (Hep3b, Hep G2, LM3 and MHCC-97H) and HEK293T cells were obtained from the American Type Culture Collection (ATCC, Manassas, VA), and were cultured in a humid atmosphere of 5% CO2 with DMEM supplemented with 10% fetal bovine serum (penicillin and streptomycin) at 37°C. All HCC cells and normal liver LO2 cells were obtained from the Institute of Biochemistry and Cell Biology, Chinese Academy of Sciences, Shanghai, China. To inhibit the expression of OLA1, we transfected three small interfering RNAs (siRNAS) targeting the OLA1 coding sequence. The siRNA sequences used are as follows: Si-1: GCTGGAA AGTACAGACAAC; Si-2: GCTGCTGGAAAGTACA GAC; and Si-3: GGGATTCATTATGGCTGAA.

The OLA1 knockout and overexpression vector, which was constructed by Shanghai Generay Biotechnology Co., Ltd., was mixed with the pPACKH1 packaging plasmid and transfected into 293TN cells. Three days later, according to the SBI instructions, the virus particles were collected via concentrated virus precipitation solution derived from *Letinus edodes*. TUNDUX virus transducers were used to infect cells. Positive cells were identified by puromycin screening.

### Cell proliferation assay, Wound-healing assay, Cell invasion assay

These methods were described in previously work and performed as indicated [44].

### Animal experiments

To establish a subcutaneous xenograft model, a total of 1 × 10^6^ LM3 and MHCC-97H cells stably expressing OLA1 knockdown (shRNA2) or negative control (NC) plasmids were resuspended in 100 μl of saline containing 50% Matrigel (BD Biosciences). The cells were subcutaneously injected into the left and right flank regions of male BALB/C nude mice aged 6 to 8 weeks. To assess tumor growth, tumor volumes were measured and recorded every 7 days using digital calipers. The mice were sacrificed on the 45th day, and the tumors were harvested, fixed, weighed, photographed and preserved.

Animal experiments were conducted under the guidance of the Institute Animal Care and Use Committee (IACUC) of Sun Yat-sen University and an application approved by the Institutional Assessment Committee of Sun Yat-sen University.

### Immunohistochemistry staining and antibodies

There were 105 formalin-fixed paraffin-embedded specimens that were used for OLA1 immunohistochemistry staining. After deparaffinization, hydration and blocking, the samples were mixed with the primary anti-OLA1 polyclonal antibody and incubated overnight at 4°C (dilution ratio 1:1,000). Finally, the HCC staining was compared and evaluated as previously described [43, 44].

### RNA extraction and quantitative real-time polymerase chain reaction (qRT-PCR)

Total RNA was isolated from tissues and cells using the Qiagen RNeasy Mini Kit in combination with on-column DNase treatment (Applied Biosystems, USA). A High Capacity RNA-to-cDNA Kit (Applied Biosystems) was used to synthesize the first strand of cDNA. Quantitative real-time PCR was performed using the Power SYBR Green PCR Master Mix (Applied Biosystems) with gene-specific primers. According to the manufacturer's instructions, the total RNA was extracted with TRIzol reagent (USA, NY, USA). qRT-PCR was performed using the SYBR Green Detection RT-PCR System (TaKaRa, Japan) using the following OLA1 primers: forward primer, ACTTTTTCACTGCAGGCCCA; reverse primer, GTACTTTCCAGCAGCCTTGAC. 18s rRNA was used as the reference control and was amplified with the following primers: forward primer, GTAACCCGTTGA ACCCCATT; reverse primer, CCATCCAATCGGTA GTAGCG. The relative mRNA expression level was determined by the 2^-ΔΔCt^ method. All qRT-PCR experiments were conducted in triplicate.

### Western blot

The tissues and cells were washed twice with 4°C PBS and then lysed in cold RIPA buffer with protease inhibitors. The BCA Protein Quantitation Assay (KeyGen Biotech, Nanjing, China) was used to measure the protein concentration. The total protein was transferred to a nitrocellulose membrane after denaturing by 10% SDS-PAGE. The membranes were blocked with 5% nonfat milk in Tris-buffered saline containing 0.1% Tween-20 (TBST) for 1 h at room temperature. The membranes were then incubated with the primary antibodies overnight at 4°C (dilution ratio 1:2,000). The membranes were washed three times with TBST and then incubated with secondary antibodies (anti-rabbit IgG or anti-mouse IgG) for 1 h at room temperature. The membranes were washed three times with TBST, and then, the targeted proteins were detected by the ECL reagent (EMD Millipore, MA, USA) method.

### Reagents

The following antibodies were purchased from Abcam: OLA1 (ab229090), GAPDH (ab8245), CCNB1 (ab71977), CCNB2 (ab18250), CCNE2 (ab40890), CDK2 (ab32147), CDK4 (ab108357), P21 (ab188224), Bcl-2 (ab32124), BAD (ab32445), cleaved caspase 3 (ab32042), Rb (ab181616), pRb (ab184796), and E2F1 (ab112580). The P21 inhibitor UC2288 was purchased from Abcam (ab146969) and used as recommended.

### Coimmunoprecipitation

Overexpressed Flag- or HA-labeled cell protein extracts were incubated with antibodies (or IgG as a control, Sigma) and binding protein A/G beads (Pierce) for 12 h at 4°C. After washing three times with IP buffer, the samples were analyzed by western blotting.

### Flow cytometry

HCC cells were harvested and fixed in 75% ethanol and stored overnight at 4°C. Then, the cells were stained with a DNA Prep Kit (Beckman Coulter, Brea, CA, USA), and the percentage of cells at different stages was determined by flow cytometry according to DNA content.

### Statistical analysis

The significance of continuous parameters presented as the mean ± SD in vivo and in vitro was determined by Student’s t-test. Fisher’s exact test was used for categorical parameters. The survival curves were assessed by Kaplan-Meier analysis and compared by the log-rank test. The independent prognostic factors for survival were identified by a multivariate analysis using the Cox proportional hazards regression model. Pearson correlation analysis was used to evaluate the relationships among OLA1 and related genes. P<0.05 was considered significant. Data analysis was performed using SPSS 22.0 software (IBM, USA). All experiments were independently repeated at least three times. The symbols *, ** and *** have been used to represent p<0.05, p<0.01 and p<0.001, respectively, in the Figures.

### Ethics approval

The study was approved by the Institutional Review Board for the Protection of Human Subjects of The First Affiliated Hospital of Sun Yat-Sen University and adhered to the tenets of the Declaration of Helsinki. Informed consent was obtained from the patients or their family members who agreed to the use of their samples in this study.

## Supplementary Material

Supplementary Figure 1

Supplementary Tables
