# Supplementary material for: Obg-like ATPase 1 (OLA1) overexpression predicts poor prognosis and promotes tumor progression by regulating P21/CDK2 in hepatocellular carcinoma
Source: Aging (Albany NY). 2020 Feb 11;12(3):3025–41. doi: 10.18632/aging.102797 (PMC7041778; doi:10.18632/aging.102797)
Supplement: Supplementary Figure 1 [file aging-12-102797-s001..pdf]

SUPPLEMENTARY FIGURE

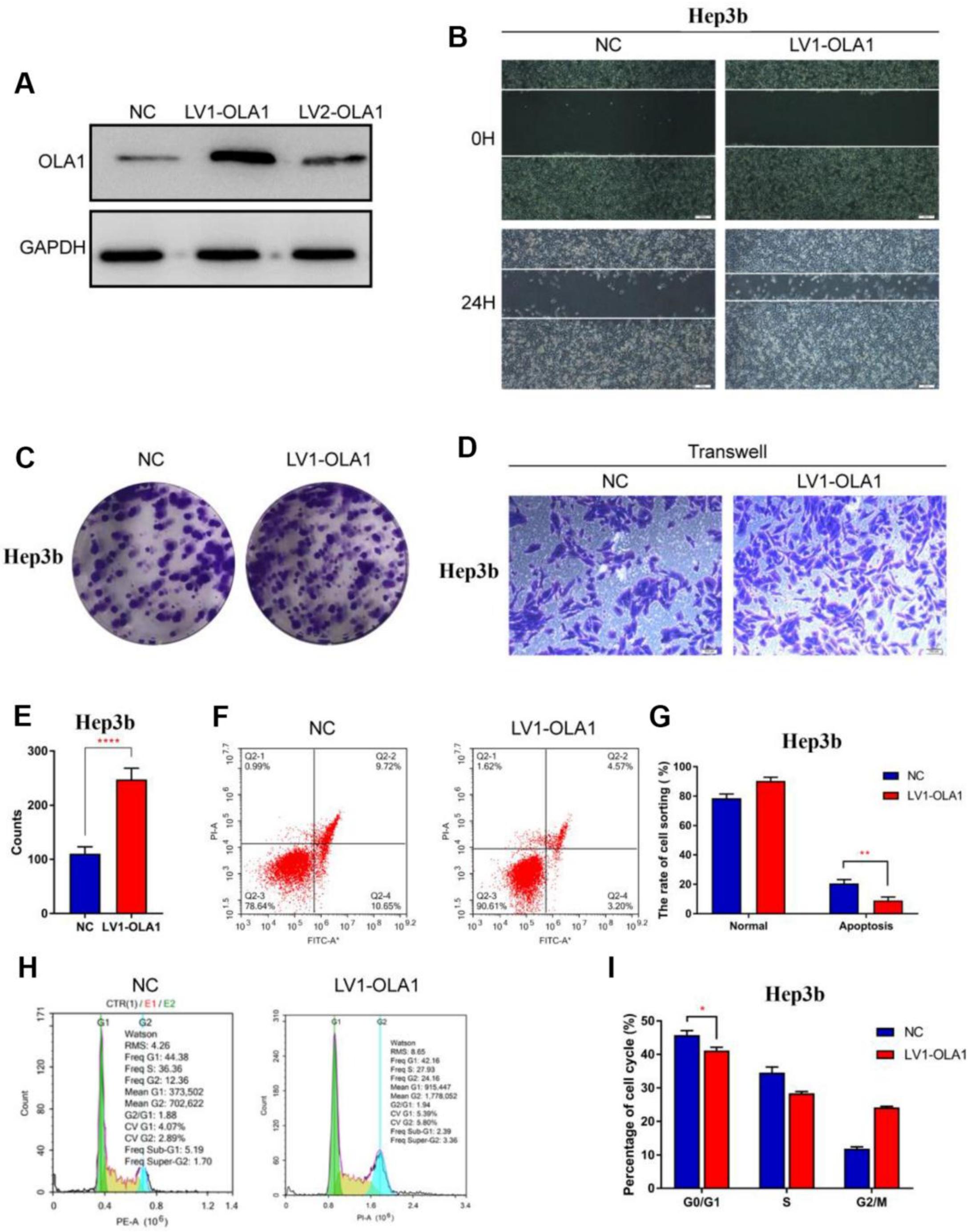

Supplementary Figure 1. Overexpression of OLA1 promotes HCC cell progression. (**A**) Overexpression of OLA1 was successfully indicated in Hep3b by western blotting. (**B**) OLA1 upregulation promoted cell migration after transfection as shown by wound healing assay. (**C**) OLA1 upregulation increased the proliferation of HCC cells as shown by colony formation assay. (**D** and **E**) The transwell Matrigel penetration assay showed that OLA1 promoted the invasion of HCC cells. (**F** and **G**) OLA1 decreases apoptosis of HCC cells ($p<0.01$). (**H** and **I**) OLA1 overexpression increased the percentage of G0/G1 phase.
