# Supplementary material for: Obg-like ATPase 1 (OLA1) overexpression predicts poor prognosis and promotes tumor progression by regulating P21/CDK2 in hepatocellular carcinoma
Source: Aging (Albany NY). 2020 Feb 11;12(3):3025–41. doi: 10.18632/aging.102797 (PMC7041778; doi:10.18632/aging.102797)
Supplement: Supplementary Tables [file aging-12-102797-s002..pdf]

SUPPLEMENTARY TABLES

Supplementary Table 1. Correlation between OLA1 expression with clinicopathological characteristics of HCC patients.

Clinicopathological variables	n	OLA1 expression		*P* Value
		Low (35)	High (70)	
Sex				
Male	90	30	60	
Female	15	5	10	0.61
Age, years				
<50	52	19	33	0.54
≥50	53	16	37	
AFP, ng/L				
<200	50	20	39	0.21
≥200	55	15	40	
HBsAg				
Negative	42	18	24	0.10
Positive	63	17	46	
Tumor size, cm				
≤5	54	29	25	**<0.01**
>5	51	6	45	
Tumor number				
Solitary	57	28	29	**0.02**
Multiple (≥2)	43	11	32	
PVTT				
Absence	69	31	38	**<0.01**
Presence	36	4	32	
TNM stage				
Early (I & II)	61	27	34	
Late (III & IV)	44	8	36	**0.01**
Differentiation grade				
Well	68	32	36	
Poor	37	3	34	**<0.01**

Abbreviation: AFP, alpha fetoprotein; HBsAg, hepatitis B surface antigen; PVTT, portal vein tumor thrombus.

Supplementary Table 2. Univariate and multivariate Cox regression analysis of risk factors associated with overall survival.

Variables	Univariate analysis			Multivariate analysis		
	HR	95% CI	*P* Value	HR	95% CI	*P* Value
OLA1 expression (High vs. Low)	7.00	3.00-16.37	**<0.01**	5.42	2.12-13.89	**<0.01**
Sex (Male vs. Female)	2.05	0.93-4.53	0.05			
Age (≥50 vs. <50)	1.10	0.67-1.79	0.72			
AFP (≥200 ng/ml vs. <200ng/ml)	1.61	0.96-2.69	0.06			
HBsAg (Positive vs. Negative)	1.34	0.79-2.26	0.27			
Tumor size (>5 cm vs. ≤5 cm)	3.60	2.08-6.22	**<0.01**	2.39	1.24-4.64	**0.01**
Tumor number (Multiple vs. Single)	1.47	0.90-2.41	0.13			
PVTT (Presence vs. Absence)	2.32	1.42-3.79	**<0.01**	1.17	0.56-2.42	0.68
TNM stage (Late vs. Early)	2.96	1.77-4.95	**<0.01**	1.47	0.71-3.02	0.30
Differentiation grade (Poor vs. Well)	2.18	1.33-3.56	**<0.01**	0.60	0.31-1.18	0.14

Abbreviation: AFP, alpha fetoprotein; HBsAg, hepatitis B surface antigen; PVTT, portal vein tumor thrombus.

Supplementary Table 3. Univariate and multivariate Cox regression analysis of risk factors associated with disease-free survival.

Variables	Univariate analysis			Multivariate analysis		
	HR	95% CI	*P* Value	HR	95% CI	*P* Value
OLA1 expression (High vs. Low)	8.53	3.39-21.47	**<0.01**	6.15	2.26-16.74	**<0.01**
Sex (Male vs. Female)	1.88	0.85-4.16	0.09			
Age (≥50 vs. <50)	1.06	0.64-1.75	0.83			
AFP (≥200 ng/ml vs. <200ng/ml)	1.64	0.97-2.77	0.06			
HBsAg (Positive vs. Negative)	1.38	0.81-2.36	0.23			
Tumor size (>5 cm vs. ≤5 cm)	4.13	2.33-7.33	**<0.01**	2.94	1.49-5.79	**<0.01**
Tumor number (Multiple vs. Single)	1.58	0.96-2.62	0.08			
PVTT (Presence vs. Absence)	2.30	1.39-3.79	**<0.01**	0.93	0.44-1.97	0.85
TNM stage (Late vs. Early)	3.30	1.94-5.62	**<0.01**	1.82	0.86-3.85	0.12
Differentiation grade (Poor vs. Well)	2.31	1.38-3.87	**<0.01**	1.79	0.90-3.57	0.10

Abbreviation: AFP, alpha fetoprotein; HBsAg, hepatitis B surface antigen; PVTT, portal vein tumor thrombus.
